# Dipyridamole augments the antiinflammatory response during human endotoxemia

**DOI:** 10.1186/cc10576

**Published:** 2011-11-30

**Authors:** Bart P Ramakers, Niels P Riksen, Thijmen H Stal, Suzanne Heemskerk, Petra van den Broek, Wilbert HM Peters, Johannes G van der Hoeven, Paul Smits, Peter Pickkers

**Affiliations:** 1 Department of Pharmacology-Toxicology, Radboud University Nijmegen Medical Center, Geert Grooteplein 10, 6500 HB, Nijmegen, The Netherlands; 2 Department of Intensive Care Medicine, Radboud University Nijmegen Medical Center, Geert Grooteplein 10, 6500 HB, Nijmegen, The Netherlands; 3 Department of Internal Medicine, Radboud University Nijmegen Medical Center, Geert Grooteplein 10, 6500 HB, Nijmegen, The Netherlands; 4 Department of Gastroenterology, Radboud University Nijmegen Medical Center, Geert Grooteplein 10, 6500 HB, Nijmegen, The Netherlands; 5 Nijmegen Institute for Infection, Inflammation and Immunity (N4i), Radboud University Nijmegen Medical Center, Geert Grooteplein 10, 6500 HB, Nijmegen, The Netherlands

## Abstract

**Introduction:**

In animal models of systemic inflammation, the endogenous nucleoside adenosine controls inflammation and prevents organ injury. Dipyridamole blocks the cellular uptake of endogenous adenosine and increases the extracellular adenosine concentration. We studied the effects of oral dipyridamole treatment on innate immunity and organ injury during human experimental endotoxemia.

**Methods:**

In a randomized double-blind placebo-controlled study, 20 healthy male subjects received 2 ng/kg *Escherichia coli *endotoxin (lipopolysaccharide; LPS) intravenously after 7-day pretreatment with dipyridamole, 200 mg slow release twice daily, or placebo.

**Results:**

Nucleoside transporter activity on circulating erythrocytes was reduced by dipyridamole with 89% ± 2% (*P *< 0.0001), and the circulating endogenous adenosine concentration was increased. Treatment with dipyridamole augmented the LPS-induced increase in the antiinflammatory cytokine interleukin (IL)-10 with 274%, and resulted in a more rapid decrease in proinflammatory cytokines tumor necrosis factor-α (TNF-α) and IL-6 levels directly after their peak level (*P *< 0.05 and < 0.01, respectively). A strong correlation was found between the plasma dipyridamole concentration and the adenosine concentration (*r *= 0.82; *P *< 0.01), and between the adenosine concentration and the IL-10 concentration (*r *= 0.88; *P *< 0.0001), and the subsequent decrease in TNF-α (*r *= -0.54; *P *= 0.02). Dipyridamole treatment did not affect the LPS-induced endothelial dysfunction or renal injury during experimental endotoxemia.

**Conclusions:**

Seven-day oral treatment with dipyridamole increases the circulating adenosine concentration and augments the antiinflammatory response during experimental human endotoxemia, which is associated with a faster decline in proinflammatory cytokines.

**Trial registration:**

ClinicalTrials (NCT): NCT01091571.

## Introduction

During sepsis, unopposed and prolonged activation of the innate immune system can induce significant collateral damage to host tissues, resulting in a high mortality rate. During inflammation, the extracellular concentration of the purine nucleoside adenosine rapidly increases [1-3]. Subsequent receptor activation acts as a physiological negative-feedback mechanism that dampens the inflammatory response [4]. Indeed, administration of adenosine-receptor agonists exerts antiinflammatory and tissue-protective effects and reduces mortality in animal models of systemic inflammation [5,6].

**Figure 1:**
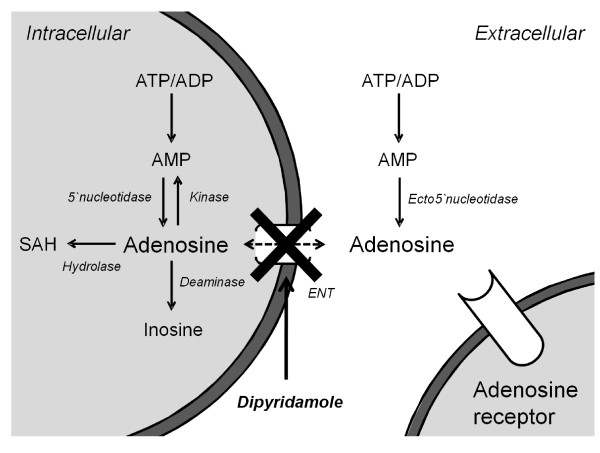
**Schematic representation of the adenosine metabolism**. Dipyridamole acts as an adenosine reuptake inhibitor through inhibition of the nucleoside transporter. ADP, adenosine diphosphate; AMP, adenosine monophosphate; ATP, adenosine triphosphate; SAH, *S*-adenosylhomocysteine.

Dipyridamole blocks the equilibrative nucleoside transporter (ENT), which facilitates the transmembranous diffusion of adenosine (Figure 1). Dipyridamole will increase the extracellular endogenous adenosine concentration, mainly in situations of increased extracellular formation of adenosine, such as occurs during hypoxia or inflammation [7]. In animals, the administration of ENT blockers attenuates LPS-induced leukopenia and tumor necrosis factor-α (TNF-α) production [8] and reduces the severity of tissue injury in several inflammatory models [9-11].

We hypothesized that dipyridamole may ameliorate the excessive and prolonged activation of the immune response that can occur during systemic inflammation. Therefore, in a proof-of-concept study in healthy volunteers, we examined whether pretreatment with dipyridamole curtails the activation of the innate immune system during experimental endotoxemia and prevents (subclinical) organ damage.

## Materials and methods

### Healthy volunteers

This study was approved by the local ethics committee and registered (http://www.clinicaltrials.gov, NCT01091571). After signing for informed consent, 20 healthy male volunteers participated. Because of significant differences in the innate immune response between male and female subjects during experimental endotoxemia, we included only male subjects [12]. All volunteers were asked not to take any drugs or caffeine-containing substances 48 hours before the start of the endotoxemia experiment. Subjects were randomized in a double-blinded fashion to 7-day pretreatment with dipyridamole (200 mg BID orally, Persantin Retard; Boehringer-Ingelheim, Alkmaar, The Netherlands; *n *= 10) or placebo (microcrystalline cellulose, *n *= 10), based on the fact that a steady state occurs after 3 days, and previous studies that examined the effects of dipyridamole during ischemia were also performed after 7 days of treatment [13]. Oral dipyridamole and placebo capsules were provided and labeled by the Department of Clinical Pharmacy of the Radboud University Nijmegen Medical Center according to GMPstandards. Both capsules had the same appearance.

### Experimental protocol

**Figure 2:**
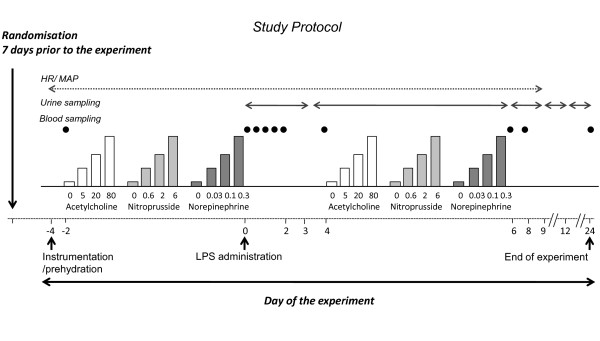
**Schematic presentation of the endotoxemia experiments**.

After local anesthesia, the brachial artery of the nondominant arm was cannulated for blood pressure monitoring, blood sampling, and administration of vasoactive drugs [14]. A second cannula was placed in a deep antecubital vein for prehydration [15]. U.S. Reference *E. coli *endotoxin (*Escherichia coli *O:113; Clinical Center Reference Endotoxin, National Institutes of Health, Bethesda, MD (LPS)) was administered as a bolus infusion in 1 minute (2 ng/kg) at *t *= 0 hours, after vortex mixing for 30 minutes. The protocol is illustrated in Figure 2.

### Analytic procedures

Plasma caffeine and dipyridamole concentrations were determined by reversed-phase high-power liquid chromatography (HPLC) [16]. Circulating adenosine concentrations were measured before and during endotoxemia, and the activity of the ENT was measured in isolated erythrocytes by measuring uridine uptake, as previously described [13,17]. Concentrations of tumor necrosis factor (TNF)-α, interleukin (IL)-6, IL-1 receptor antagonist (IL1ra), IL-10, intercellular adhesion molecule 1 (ICAM-1), and vascular adhesion molecule 1 (VCAM-1) were analyzed in batches by using a Luminex assay (Bio-plex cytokine assay; BioRad, Hercules, CA, USA). The antioxidant capacity in blood plasma was measured by using the ferric reducing ability of plasma (FRAP) assay, according to the method of Benzie and Strain [18].

### Forearm blood-flow measurements

Forearm blood flow (FBF, milliliters per minute per deciliter forearm volume) was measured 2 hours before and 4 hours after LPS administration with venous occlusion plethysmography (Filtrass Domed, Munich, Germany) [19]. The vasodilator response to intrabrachial infusion of acetylcholine (5, 20, 80 μg/min/dl) and nitroprusside (0.6, 2, 6 μg/min/dl), and the vasoconstrictor response to norepinephrine (0.03, 0.1, 0.3 μg/min/dl) was quantified [20]. Infusion rates of drugs and measurements of forearm blood volume were normalized to forearm blood volume and expressed per deciliter of forearm volume.

### Drugs and solutions

Acetylcholine (Novartis Pharma, Nurnberg, Germany) and norepinephrine (Centrafarm BV, Etten-Leur, The Netherlands) were dissolved in normal saline, and nitroprusside (Clinical Pharmacy, Radboud University Nijmegen Medical Centre) was dissolved in a 5% glucose solution. All solutions were freshly prepared at the day of the experiment.

### Urine collection

Subjects collected their morning urine before treatment with dipyridamole or placebo and on the day of the LPS experiment. After start of the LPS infusion, urine was collected in four 3-hour periods and during a period of 12 to 24 hours (see Figure 2). During the sampling period, urine was kept on ice. Urine volume was measured, and creatinine, glutathione *S*-transferase (GST) alpha (A1-1) and pi (P1-1), as markers of proximal and distal tubule injury, respectively, were measured [21].

### Statistical analyses

The effect of dipyridamole was analyzed by using a repeated measures analysis of variance (ANOVA), with *post hoc *tests for specific time points (Bonferroni). Further to substantiate the possible mechanism of action of dipyridamole, Pearson correlations were conducted to explore the correlation between plasma levels of dipyridamole, adenosine, and cytokines. The lines were calculated from linear regression analyses.

The area under the curve (AUC) of the increase in FBF was calculated (before and after LPS administration). The LPS-mediated difference was compared between groups by using an unpaired Student *t *test. The effect of endotoxemia on FRAP was tested by using a repeated measures ANOVA. Because data had a gaussian distribution, data are expressed as mean ± SEM, unless specified otherwise. Nonparametric data are illustrated as box-and-whiskers. A *P *value < 0.05 was considered statistically significant.

## Results

### Demographic characteristics

The demographic characteristics were comparable between groups (Table 1). Plasma caffeine concentrations immediately before LPS administration were < 0.06 mg/L in both the dipyridamole and the placebo groups. The incidence of side effects was not significantly different between the dipyridamole and the placebo groups.

**Table 1:** Demographic characteristics

	Placebo (*n *= 10)	Dipyridamole (*n *= 10)
Age (years)	21.4 ± 1.8	22 ± 2.6
Height (m)	1.86 ± 0.1	1.84 ± 0.1
Weight (kg)	84.4 ± 10.6	75.8 ± 8.5
BMI (kg/m^2^)	24.5 ± 3.9	22.3 ± 1.9
Heart rate (beats per minute)	62 ± 7	60 ± 5
MAP (mm Hg)	96 ± 4	91 ± 7
Forearm volume (ml)	1,190 ± 124	1,033 ± 96

Data reported as mean ± SD.

### Effect of dipyridamole on circulating adenosine

The plasma dipyridamole concentration at the moment of LPS administration (*t *= 0) averaged 1.8 ± 0.3 and 0.0 ± 0.0 mg/L for the dipyridamole and placebo groups, respectively.

Uridine uptake into the erythrocyte via the ENT was profoundly inhibited by dipyridamole: from 113 ± 9 nmol/10^9 ^erythrocytes/min at baseline to 11 ± 2 nmol/10^9 ^erythrocytes/min immediately before the LPS experiment (*P *< 0.0001). In placebo-treated subjects, uridine transport was 112 ± 7 nmol/10^9 ^erythrocytes/min at baseline and 124 ± 7 nmol/10^9 ^erythrocytes/min immediately before the LPS experiment (*P *= 0.86).

**Figure 3:**
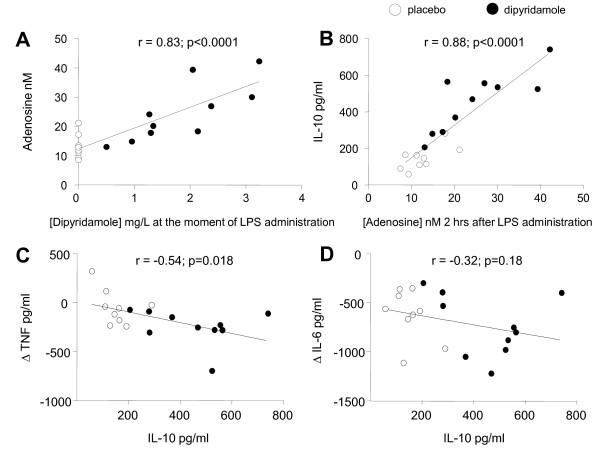
**Correlations between (a) the dipyridamole concentration at the moment of LPS administration and the (peak) adenosine concentration, 2 hours after LPS administration; (b) the peak adenosine concentration and peak IL-10 levels; (c) peak IL-10 concentrations and the decline in TNF-α levels (*t *= 2 to *t *= 90 minutes after LPS administration); and (d) peak IL-10 concentrations and the decline in IL-6 levels**.

Seven-day treatment with dipyridamole resulted in a higher adenosine concentration before the LPS administration; 22.6 ± 2.7 nmol/ml compared with 11.1 ± 1.8 nmol/ml in the placebo group (*P *< 0.01). The adenosine concentration further increased with 2.1 ± 2.8 and 2.1 ± 0.9 nmol/ml after administration of LPS in both groups (*P *= 0.99, difference between both groups). Dipyridamole concentrations correlated strongly with peak adenosine concentrations (*r *= 0.82; *P *< 0.01, see Figure 3a).

### Innate immune response

#### Inflammatory parameters during human endotoxemia

During the first hour after LPS administration, the total white blood cell count decreased from 6.2 ± 0.3 to 2.2 ± 0.3 × 10^9^/L and from 5.7 ± 0.6 to 2.2 ± 0.3 × 10^9^/L for dipyridamole- and placebo-treated subjects, after which there was an increase to 13.6 ± 0.7 × 10^9^/L and 11.9 ± 0.6 × 10^9^/L at 8 hours after LPS (*P *= 0.07). Dipyridamole-treated subjects had significantly higher amounts of circulating monocytes in the period of 4 to 8 hours after LPS, with a peak at 8 hours after LPS administration (0.64 ± 0.08 × 10^9^/L in dipyridamole-treated subjects versus 0.37 ± 0.03 × 10^9^/L in placebo; *P *= 0.04). The increase in body temperature after administration of LPS was similar in the dipyridamole and placebo groups; from 36.5°C ± 0.1°C to 38.0°C ± 0.2°C and from 36.4°C ± 0.1°C to 38.2°C ± 0.1°C, respectively (*P *= 0.76 between groups).

**Figure 4:**
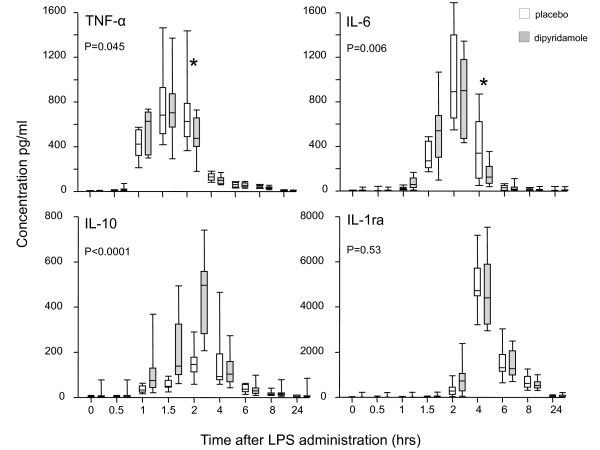
**Box-and-whiskers (whiskers, range) of the cytokine response after LPS administration in placebo-treated subjects (open symbols) and dipyridamole-treated subjects (solid symbols), *n *= 10 subjects per group**. The probability values refer to the statistical difference between the placebo- and dipyridamole-treated groups in response to LPS administration, as analyzed with a two-way ANOVA. *P < 0.05 between groups, as analyzed with a Bonferroni posttest.

Dipyridamole treatment augmented the IL-10 response during endotoxemia (*P *< 0.0001 compared with the placebo group; Figure 4). Moreover, the endogenous adenosine concentration 2 hours after LPS administration correlated with peak levels of IL-10 (*r *= 0.88; *P *< 0.0001), as illustrated in Figure 3b. The LPS-induced peak concentrations of proinflammatory cytokines were not influenced by dipyridamole treatment. In contrast, the decline of TNF-α and IL-6 levels directly after their highest value was accelerated in dipyridamole-treated subjects (*P *< 0.05 and < 0.01, respectively; Figure 4). The peak IL-10 levels correlated with the decline of TNF-α (*r *= -0.54; *P *= 0.02), but not with that of IL-6 (*r *= -0.32; *P *= 0.18), Figure 3c and 3d.

### LPS-induced end-organ dysfunction

#### Clinical and hemodynamic parameters during human endotoxemia

In all volunteers, LPS administration induced the expected flu-like symptoms. Experimental endotoxemia resulted in a vasodilatory state, illustrated by a decrease in blood pressure and an increase in heart rate and forearm blood flow, with a maximum effect at *t *= 4 to 6 hours after LPS administration (Table 2). This LPS-induced cardiovascular response was similar between groups.

**Table 2:** Hemodynamic profile in response to endotoxemia

	Placebo		Dipyridamole		Difference between groups
	Baseline	4 to 6 hours after LPS	Baseline	4 to 6 hours after LPS	(*P *value)
HR (beats per minute)	65 ± 2	84 ± 2	67 ± 2	89 ± 1	0.59
SBP (mm Hg)	132 ± 1	121 ± 2	134 ± 2	114 ± 2	0.29
DBP (mm Hg)	76 ± 1	71 ± 2	74 ± 1	68 ± 1	0.6
MAP (mm Hg)	94 ± 1	87 ± 2	94 ± 1	83 ± 1	0.35
FBF ml/min/dl	2.7 ± 0.4	7.3 ± 0.8	4.3 ± 0.5	8.6 ± 1.2	0.87

Data expressed as mean ± SEM. DBP, diastolic blood pressure; FBF, forearm blood flow; HR, heart rate; MAP, mean arterial pressure; SBP, systolic blood pressure. All LPS-induced changes were significant (*P *< 0.0001).

#### FBF response to acetylcholine, nitroprusside and norepinephrine

**Figure 5:**
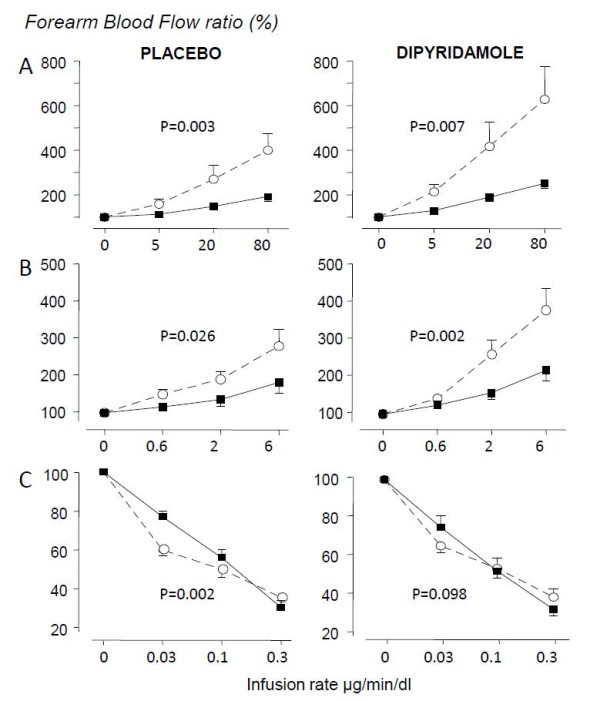
**Dose-response curve of intrabrachial infusion of (a) acetylcholine, (b) nitroprusside, and (c) norepinephrine on forearm blood flow (FBF) before (open symbols, dotted line) and 4 hours after administration of 2 ng/kg *Escherichia coli *LPS (solid symbols)**. Data are presented as percentages of baseline FBF of the intervention arm (mean ± SEM; *n *= 10 per group). Left panel shows placebo-treated subjects; right panel, subjects treated with dipyridamole. The probability values refer to the statistical difference between the dose-response curves, as analyzed with two-way ANOVA.

Subjects treated with dipyridamole had a lower baseline FBF (2.7 ± 0.4 versus 4.3 ± 0.5 ml/min/dl in placebo-treated participants, *P *= 0.03). This baseline difference disappeared after LPS administration: 7.3 ± 0.8 in the dipyridamole group versus 8.6 ± 1.2 ml/min/dl in the placebo group. No significant changes in FBF were found in the noninfused forearm during the intrabrachial infusions of acetylcholine, nitroprusside, and norepinephrine, excluding systemic hemodynamic effects of these drugs. As shown in Figure 5, endothelium-dependent (a) and independent vasodilatation (b) as well as norepinephrine-induced vasoconstriction (c) were significantly impaired after endotoxemia. No significant differences were noted between the treatment groups.

#### Circulating adhesion molecules

Baseline plasma levels of ICAM and VCAM tended to be higher in dipyridamole-treated subjects (ICAM: dipyridamole, 12.0 ± 0.7 × 10^4 ^pg/ml, versus placebo, 10.0 ± 0.7 × 10^4 ^pg/ml (*P *= 0.05); VCAM: dipyridamole, 19.1 ± 1.2 × 10^4 ^pg/ml versus placebo, 16.5 ± 0.7 × 10^4 ^pg/ml (*P *= 0.08)). Both ICAM and VCAM levels increased after LPS administration (*P *< 0.0001), but dipyridamole treatment did not affect the endotoxemia-induced increase in ICAM and VCAM levels (difference between groups: *P *= 0.31 and *P *= 0.90, respectively).

#### Oxidative stress

**Figure 6:**
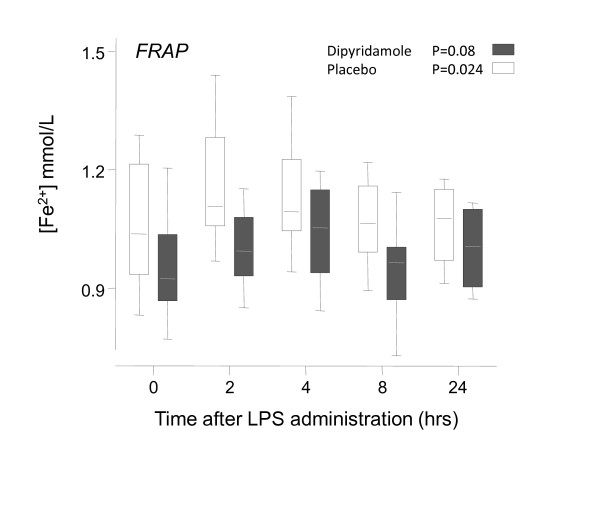
**Box-and-whiskers (whiskers, range) of the endotoxemia-induced changes in FRAP**. FRAP increases during endotoxemia, in both dipyridamole- and placebo-treated subjects (*P *= 0.08 and 0.02, respectively). No differences between groups were found, as analyzed with two-way ANOVA (*P *= 036).

The total antioxidant capacity, as measured with FRAP, increased during the first 2 hours after endotoxemia from 0.96 ± 0.04 to 1.00 ± 0.03 mmol/L and from 1.06 ± 0.05 to 1.16 ± 0.05 mmol/L (*P *= 0.08 and *P *= 0.02 for dipyridamole and placebo groups, respectively). No significant difference in FRAP was found between both groups (*P *= 0.36; Figure 6).

#### Renal injury

Endotoxemia resulted in a cumulative GSTA1-1 excretion of 11.2 (6.2 to 13.0) μg compared with 5.1 (3.9 to 9.4) μg 12 hours after LPS administration in dipyridamole- and placebo-treated subjects, respectively. Cumulative GSTP1-1excretion was 6.4 (5.4 to 7.8) μg and 6.3 (4.5 to 8.0) μg, respectively. No differences were seen between the LPS-induced increase between both groups (*P *= 0.07 and *P *= 0.44, respectively).

## Discussion

In the current study, we showed for the first time in humans *in vivo *that oral treatment with the nucleoside transport inhibitor dipyridamole augments the anti-inflammatory response of the innate immune system during experimental endotoxemia. Treatment with dipyridamole effectively blocked nucleoside uptake and resulted in a significant increase in the circulating endogenous adenosine concentration. In the dipyridamole-treated subjects, the antiinflammatory IL-10 response to LPS administration was highly augmented, and was associated with an accelerated decline of the proinflammatory cytokines TNF-α and IL-6 after their initial increase. We demonstrated that dipyridamole concentrations correlated with adenosine concentrations, that higher adenosine concentrations were associated with higher IL-10 concentrations, and that higher IL-10 levels were associated with a more pronounced decline of TNF-α. These subsequent correlations suggest that the immunomodulating effects of dipyridamole are mediated through the adenosine pathway.

The purine nucleoside adenosine is a well-known endogenous signaling molecule with potent antiinflammatory and tissue-protective properties [1,22,23]. During systemic inflammation, the endogenous adenosine concentration rapidly increases [3,24], with circulating concentrations doubled during experimental human endotoxemia [17] and increasing up to tenfold in septic shock patients [3]. However, interpretation of these measurements must be addressed with caution, because adenosine measurement is notoriously troublesome [25]. Subsequent stimulation of membrane-bound adenosine receptors may act as a negative-feedback mechanism to control and curtail the inflammatory response and to attenuate further organ damage. Indeed, animal studies previously showed that adenosine plays a pivotal role in the protection of tissue against damage from excessive inflammation (for example, during sepsis [4,5]). In addition, the administration of adenosine-receptor agonists potently limits inflammation in murine models of systemic inflammation [5,6]. Human data on the role of adenosine during systemic inflammation are scarce. Continuous intravenous administration of adenosine attenuated the IL-6 response during human endotoxemia [26]. However, therapeutic administration of adenosine is cumbersome, because of the extremely short half-life of adenosine, the hemodynamic effects during systemic administration, and because the endothelium acts as a strong metabolic barrier for adenosine, preventing adenosine from entering the interstitial compartment [27]. As such, the plasma concentration of adenosine does not represent tissue interstitial concentrations of adenosine. This is relevant, because resident tissue macrophages appear to be the major source of circulating cytokines. By preventing cellular uptake of adenosine, dipyridamole increases the endogenous extracellular adenosine concentration mainly in those tissues where extracellular adenosine formation is increased (that is, at the site of inflammation). Therefore, we hypothesized that dipyridamole controls inflammation more effectively than does exogenous adenosine, with fewer hemodynamic side effects.

Our results are in accordance with previous *in vitro *and animal experiments on the immunomodulating effect of nucleoside transport inhibition. Dipyridamole enhances the LPS-induced IL-10 production [28] and attenuates the production of TNF-α [29] and other proinflammatory cytokines in human cultured mononuclear cells. Furthermore, dipyridamole therapy in patients undergoing coronary artery bypass grafting inhibited postoperative *ex vivo *polymorphonuclear cell adhesion to endothelial cells [30]. Also in animal studies, administration of ENT inhibitors limited the inflammatory response and reduced tissue injury in situations of severe inflammation [9-11]. Of importance, these effects were abolished by concomitant administration of adenosine A_2a _receptor antagonists [31].

The immune-modulating effects of dipyridamole are sparsely studied in humans *in vivo*. We have previously shown that dipyridamole reduces ischemia-reperfusion injury in healthy volunteers [13]. To our knowledge, apart from a small study in patients with rheumatoid arthritis [32], dipyridamole has never been tested in situations of generalized inflammation. In this latter study, dipyridamole was not found to reduce inflammation [32], but a clear conclusion is not possible, as it appears likely that this study was underpowered.

In our study, treatment with dipyridamole profoundly enhanced the antiinflammatory IL-10 response during endotoxemia. IL-10 is produced by cells of the innate immune system and is able to inhibit the synthesis of various proinflammatory cytokines, including TNF-α, in an autoregulatory fashion [33]. In accordance, administration of IL-10 protects mice from lethal endotoxemia [34], and IL-10 knockout mice have a more-pronounced hemodynamic response to LPS administration [35]. Given the strong association between the plasma adenosine and IL-10 concentration, we propose that dipyridamole augments the IL-10 response by increasing the endogenous adenosine concentration. Indeed, animal studies have shown that adenosine-receptor agonists augment the IL-10 response to LPS [6]. In accordance with the antiinflammatory role of IL-10, we observed a more-rapid decrease in plasma TNF-α and IL-6 after the peak concentrations of these proinflammatory cytokines. In contrast, peak plasma levels of TNF-α and IL-6 were not affected by dipyridamole. It appears plausible that this initial proinflammatory response is needed as a stimulus for increased adenosine formation at the site of inflammation. Dipyridamole may therefore enhance the antiinflammatory properties of adenosine only directly after the initial proinflammatory insult. In our study, dipyridamole increased the baseline plasma adenosine concentration, but did not augment the LPS-induced increase in adenosine. This may be explained by the fact that adenosine can be highly compartmentalized, as described earlier.

Despite the observation that treatment with dipyridamole modulates the plasma cytokine response during endotoxemia toward a more antiinflammatory profile, this treatment did not prevent LPS-induced vascular dysfunction and renal injury, nor did it influence the LPS-induced increase in FRAP. Our observation that endotoxemia increases FRAP concentrations is in accordance with a previously described FRAP increase observed in sepsis patients [36]. We postulate that this lack of an effect on organ injury is due to the relatively mild and short-lasting inflammatory insult induced during experimental endotoxemia, and it may not rule out the possibility that the antiinflammatory effects of dipyridamole prevent organ dysfunction in the setting of a more severe or more persistent proinflammatory insult, such as during sepsis or autoimmune diseases.

Given the fact that dipyridamole treatment has limited side effects and modulates the innate immune response to a relevant extent, further studies are warranted to explore the immunomodulating potential in patients with systemic inflammation.

## Conclusions

Seven-day oral treatment with dipyridamole is associated with increased circulating levels of adenosine and an augmented antiinflammatory response during human experimental endotoxemia that may curtail the release of proinflammatory cytokines.

## Key messages

◆ Seven-day treatment with dipyridamole increases the endogenous adenosine concentration and augments the antiinflammatory response during human experimental endotoxemia.

◆ A strong correlation exists between the dipyridamole concentration and the endogenous adenosine concentration, which in turn correlates with the IL-10 response.

◆ The more-pronounced increase in IL-10 is associated with an accelerated decline of proinflammatory cytokines.

◆ Immunomodulating properties of dipyridamole may be of therapeutic benefit in patients with severe or persisting systemic inflammation.

## Abbreviations

ANOVA: analysis of variance; FRAP: ferric reducing ability of plasma; GSTA1-1: glutathione *S*-transferase alpha 1-1; GSTP1-1: glutathione *S*-transferase pi 1-1; ICAM: intercellular adhesion molecule; IL: interleukin; IL1RA: interleukin-1-receptor antagonist; IQR: interquartile range; LPS: lipopolysaccharide; TNF-α: tumor necrosis factor-alpha; VCAM: vascular cell-adhesion molecule.

## Competing interests

The authors declare that they have no competing interests.

## Authors' contributions

BPR and THS carried out the study, and BPR gathered all data, performed the statistical analysis, and wrote the manuscript. PvdB performed the adenosine, dipyridamole, and caffeine measurements. SH and WHMP performed the GSTA1-1, GSTP1-1, and FRAP analyses. PP, NPR, and PS supervised the conduct of the study and the writing of the paper. JGvdH corrected the manuscript. All authors read and approved the final manuscript.

## Authors' information

BPR is a recipient of an AGIKO fellowship, and NPR is a recipient of a clinical fellowship, both of the Netherlands Organization for Health Research and Development (ZonMw).

## References

[B1] Hasko G Cronstein BN Adenosine: an endogenous regulator of innate immunity Trends Immunol 2004 25 33 39 10.1016/j.it.2003.11.003 14698282

[B2] Jabs CM Sigurdsson GH Neglen P Plasma levels of high-energy compounds compared with severity of illness in critically ill patients in the intensive care unit Surgery 1998 124 65 72 10.1016/S0039-6060(98)70076-5 9663253

[B3] Martin C Leone M Viviand X Ayem ML Guieu R High adenosine plasma concentration as a prognostic index for outcome in patients with septic shock Crit Care Med 2000 28 3198 3202 10.1097/00003246-200009000-00014 11008982

[B4] Ohta A Sitkovsky M Role of G-protein-coupled adenosine receptors in downregulation of inflammation and protection from tissue damage Nature 2001 414 916 920 10.1038/414916a 11780065

[B5] Sullivan GW Fang G Linden J Scheld WM A2A adenosine receptor activation improves survival in mouse models of endotoxemia and sepsis J Infect Dis 2004 189 1897 1904 10.1086/386311 15122527

[B6] Moore CC Martin EN Lee GH Obrig T Linden J Scheld WM An A2A adenosine receptor agonist, ATL313, reduces inflammation and improves survival in murine sepsis models BMC Infect Dis 2008 8 141 10.1186/1471-2334-8-141 18937852 PMC2588444

[B7] Bodin P Burnstock G Increased release of ATP from endothelial cells during acute inflammation Inflamm Res 1998 47 351 354 10.1007/s000110050341 9754870

[B8] Noji T Takayama M Mizutani M Okamura Y Takai H Karasawa A Kusaka H KF24345, an adenosine uptake inhibitor, suppresses lipopolysaccharide-induced tumor necrosis factor-alpha production and leukopenia via endogenous adenosine in mice J Pharmacol Exp Ther 2002 300 200 205 10.1124/jpet.300.1.200 11752117

[B9] Noji T Nan-ya K Katagiri C Mizutani M Sano J Nishikawa S Karasawa A Kusaka H Adenosine uptake inhibition ameliorates cerulein-induced acute pancreatitis in mice Pancreas 2002 25 387 392 10.1097/00006676-200211000-00011 12409834

[B10] Noji T Nan-ya K Mizutani M Katagiri C Sano J Takada C Nishikawa S Karasawa A Kusaka H KF24345, an adenosine uptake inhibitor, ameliorates the severity and mortality of lethal acute pancreatitis via endogenous adenosine in mice Eur J Pharmacol 2002 454 85 93 10.1016/S0014-2999(02)02476-7 12409009

[B11] Noji T Sato H Sano J Nishikawa S Kusaka H Karasawa A Treatment with an adenosine uptake inhibitor attenuates glomerulonephritis in mice Eur J Pharmacol 2002 449 293 300 10.1016/S0014-2999(02)02039-3 12167472

[B12] van Eijk LT Dorresteijn MJ Smits P van der Hoeven JG Netea MG Pickkers P Gender differences in the innate immune response and vascular reactivity following the administration of endotoxin to human volunteers Crit Care Med 2007 35 1464 1469 10.1097/01.CCM.0000266534.14262.E8 17452928

[B13] Riksen NP Oyen WJ Ramakers BP van den Broek PH Engbersen R Boerman OC Smits P Rongen GA Oral therapy with dipyridamole limits ischemia-reperfusion injury in humans Clin Pharmacol Ther 2005 78 52 59 10.1016/j.clpt.2005.03.003 16003293

[B14] Pickkers P Dorresteijn MJ Bouw MP van der Hoeven JG Smits P In vivo evidence for nitric oxide-mediated calcium-activated potassium-channel activation during human endotoxemia Circulation 2006 114 414 421 10.1161/CIRCULATIONAHA.105.590232 16864730

[B15] Dorresteijn MJ van Eijk LT Netea MG Smits P van der Hoeven JG Pickkers P Iso-osmolar prehydration shifts the cytokine response towards a more anti-inflammatory balance in human endotoxemia J Endotoxin Res 2005 11 287 293 16263001 10.1179/096805105X58715

[B16] Schreiber-Deturmeny E Bruguerolle B Simultaneous high-performance liquid chromatographic determination of caffeine and theophylline for routine drug monitoring in human plasma J Chromatogr B Biomed Appl 1996 677 305 312 10.1016/0378-4347(95)00383-5 8704934

[B17] Ramakers BP Riksen NP van den Broek P Franke B Peters WH van der Hoeven JG Smits P Pickkers P Circulating adenosine increases during human experimental endotoxemia but blockade of its receptor does not influence the immune response and subsequent organ injury Crit Care 2011 15 R3 10.1186/cc9400 21211004 PMC3222030

[B18] Benzie IF Strain JJ The ferric reducing ability of plasma (FRAP) as a measure of "antioxidant power": the FRAP assay Anal Biochem 1996 239 70 76 10.1006/abio.1996.0292 8660627

[B19] Leslie SJ Attina T Hultsch E Bolscher L Grossman M Denvir MA Webb DJ Comparison of two plethysmography systems in assessment of forearm blood flow J Appl Physiol 2004 96 1794 1799 14752120 10.1152/japplphysiol.00567.2002

[B20] Pleiner J Heere-Ress E Langenberger H Sieder AE Bayerle-Eder M Mittermayer F Fuchsjager-Mayrl G Bohm J Jansen B Wolzt M Adrenoceptor hyporeactivity is responsible for *Escherichia coli *endotoxin-induced acute vascular dysfunction in humans Arterioscler Thromb Vasc Biol 2002 22 95 100 10.1161/hq0102.101818 11788467

[B21] Heemskerk S Pickkers P Bouw MP Draisma A van der Hoeven JG Peters WH Smits P Russel FG Masereeuw R Upregulation of renal inducible nitric oxide synthase during human endotoxemia and sepsis is associated with proximal tubule injury Clin J Am Soc Nephrol 2006 1 853 862 10.2215/CJN.00490206 17699297

[B22] Fredholm BB Adenosine, an endogenous distress signal, modulates tissue damage and repair Cell Death Differ 2007 14 1315 1323 10.1038/sj.cdd.4402132 17396131

[B23] Kumar V Sharma A Adenosine: an endogenous modulator of innate immune system with therapeutic potential Eur J Pharmacol 2009 616 7 15 10.1016/j.ejphar.2009.05.005 19464286

[B24] Jabs CM Sigurdsson GH Neglen P Plasma levels of high-energy compounds compared with severity of illness in critically ill patients in the intensive care unit Surgery 1998 124 65 72 10.1016/S0039-6060(98)70076-5 9663253

[B25] Ramakers BP Riksen NP van der Hoeven JG Smits P Pickkers P Modulation of innate immunity by adenosine receptor stimulation Shock 2011 36 208 215 10.1097/SHK.0b013e318225aee4 21617576

[B26] Soop A Johansson C Hjemdahl P Kristiansson M Gyllenhammar H Li N Sollevi A Adenosine treatment attenuates cytokine interleukin-6 responses to endotoxin challenge in healthy volunteers Shock 2003 19 503 507 10.1097/01.shk.0000051756.08171.11 12785003

[B27] Gamboa A Ertl AC Costa F Farley G Manier ML Hachey DL Diedrich A Biaggioni I Blockade of nucleoside transport is required for delivery of intraarterial adenosine into the interstitium: relevance to therapeutic preconditioning in humans Circulation 2003 108 2631 2635 10.1161/01.CIR.0000101927.70100.41 14623808

[B28] Poturoglu S Kaymakoglu S Gurel PN Ibrisim D Ahishali E Akyuz F Badur S Demir K Mungan Z A new agent for tumour necrosis factor-alpha inhibition: in vitro effects of dipyridamole in Crohn's disease Scand J Clin Lab Invest 2009 69 696 702 19452347 10.3109/00365510902989075

[B29] Le V Chen YL Masson I De SM Giroud JP Florentin I Chauvelot-Moachon L Inhibition of human monocyte TNF production by adenosine receptor agonists Life Sci 1993 52 1917 1924 10.1016/0024-3205(93)90632-D 8505858

[B30] Chello M Mastroroberto P Malta E Cirillo F Celi V Inhibition by dipyridamole of neutrophil adhesion to vascular endothelium during coronary bypass surgery Ann Thorac Surg 1999 67 1277 1282 10.1016/S0003-4975(99)00173-3 10355396

[B31] Carrier EJ Auchampach JA Hillard CJ Inhibition of an equilibrative nucleoside transporter by cannabidiol: a mechanism of cannabinoid immunosuppression Proc Natl Acad Sci USA 2006 103 7895 7900 10.1073/pnas.0511232103 16672367 PMC1472541

[B32] Forrest CM Stoy N Stone TW Harman G Mackay GM Oxford L Darlington LG Adenosine and cytokine levels following treatment of rheumatoid arthritis with dipyridamole Rheumatol Int 2006 27 11 17 10.1007/s00296-006-0212-6 17021714

[B33] Saraiva M O'Garra A The regulation of IL-10 production by immune cells Nat Rev Immunol 2010 10 170 181 10.1038/nri2711 20154735

[B34] Howard M Muchamuel T Andrade S Menon S Interleukin 10 protects mice from lethal endotoxemia J Exp Med 1993 177 1205 1208 10.1084/jem.177.4.1205 8459215 PMC2190971

[B35] Hickey MJ Issekutz AC Reinhardt PH Fedorak RN Kubes P Endogenous interleukin-10 regulates hemodynamic parameters, leukocyte-endothelial cell interactions, and microvascular permeability during endotoxemia Circ Res 1998 83 1124 1131 9831707 10.1161/01.res.83.11.1124

[B36] Andresen M Regueira T Bruhn A Perez D Strobel P Dougnac A Marshall G Leighton F Lipoperoxidation and protein oxidative damage exhibit different kinetics during septic shock Mediators Inflamm 2008 2008 168652 18566692 10.1155/2008/168652 PMC2430274

